# The leading-edge vortex on a rotating wing changes markedly beyond a certain central body size

**DOI:** 10.1098/rsos.172197

**Published:** 2018-07-11

**Authors:** Shantanu S. Bhat, Jisheng Zhao, John Sheridan, Kerry Hourigan, Mark C. Thompson

**Affiliations:** *FLAIR*, Department of Mechanical and Aerospace Engineering, Monash University, Clayton, Victoria 3800, Australia

**Keywords:** insect body size, wing–body interaction, rotating wing, scanning PIV, dual-LEVs

## Abstract

Stable attachment of a leading-edge vortex (LEV) plays a key role in generating the high lift on rotating wings with a central body. The central body size can affect the LEV structure broadly in two ways. First, an overall change in the size changes the Reynolds number, which is known to have an influence on the LEV structure. Second, it may affect the Coriolis acceleration acting across the wing, depending on the wing-offset from the axis of rotation. To investigate this, the effects of Reynolds number and the wing-offset are independently studied for a rotating wing. The three-dimensional LEV structure is mapped using a scanning particle image velocimetry technique. The rapid acquisition of images and their correlation are carefully validated. The results presented in this paper show that the LEV structure changes mainly with the Reynolds number. The LEV-split is found to be only minimally affected by changing the central body radius in the range of small offsets, which interestingly includes the range for most insects. However, beyond this small offset range, the LEV-split is found to change dramatically.

## 1. Introduction

Further advances in the design of micro air vehicles (MAVs) may require detailed understanding of the aerodynamics of the flapping wings of insects, which outperform the lifting mechanisms used in standard MAVs. Researchers in the past have proposed different mechanisms in order to explain the higher lift observed for insects wings flapping at very high angles of attack.

The stable attachment of the leading-edge vortex (LEV) at such high angles of attack (*α*∼45°), as observed by Maxworthy [1] and Ellington *et al.* [2], is considered to be the principal mechanism. The LEV is formed and sustained during a major part of the flapping stroke in which the wing maintains a high angle of attack (*α*∼45°) while rotating at a constant angular velocity. Thus, studies of a purely rotating wing model can provide insights into flapping wing aerodynamics. When a wing rotates with such a high angle, the flow separates near the leading edge, forming a separating shear layer. In turn, this shear layer rolls up to form the LEV.

The LEV remains attached to the wing throughout its rotation without changing its size. Ellington *et al.* [2] attributed the reason behind its stability over a hawkmoth wing flapping at a high Reynolds number (*Re*∼5000) to the spanwise axial flow and the delayed stall, whereas Birch *et al.* [3] attributed it to the reduction in downwash angle owing to the tip vortex on a fruit fly wing planform rotating at a low Reynolds number (*Re* = 160). Shyy & Liu [4] compared the low and high Reynolds number flows over a flapping insect wing and concluded that the weaker swirl at low Reynolds numbers is responsible in maintaining the LEV structure. More recently, Nabawy & Crowther [5] have investigated the role of the LEV in lift augmentation and proposed that the absence of stall over a rotating wing could explain the improved performance and the additional lift. Bomphrey *et al.* [6] have discussed various LEV topologies, namely, (i) single continuous LEV spanning both the wings and the thorax; (ii) separate LEVs on both the wings; and (iii) separate LEVs leading into the respective root and tip vortex systems. The second category is likely to occur in mechanical models and also in some real insects, which is observed and discussed here.

**Figure 1.**
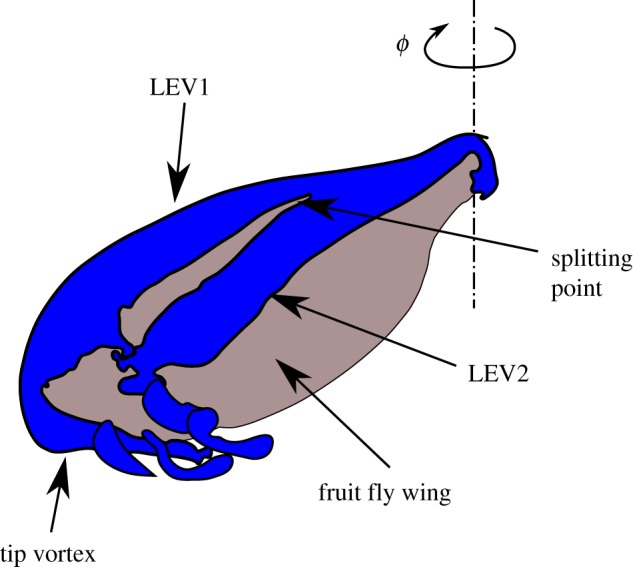
The schematic adopted from Harbig *et al.* [9] shows the dual-LEVs formed over a rotating fruit fly wing.

Fluid motion within the LEV is helical in nature, growing in size from the wing-root to the wing-tip [7]. This growth is accompanied by an increase in the strength of the sectional circulation. Above a minimum Reynolds number, at a certain point along the span, the LEV splits into two co-rotating vortex cores. This structure is often referred to as dual-LEVs. Lu *et al.* [8] have attributed the LEV split to the outboard separation caused by boundary-layer induced secondary vorticity generated while the primary shear layer rolls up to form the major vortex (LEV2). Figure 1 shows a schematic of the dual-LEVs formed over a rotating fruit fly wing. While the vorticity in LEV2 is transported towards the wing tip through axial flow, the separating shear layer continues to feed vorticity into the minor vortex (LEV1) closer to the leading edge. The LEVs merge with the tip-vortex leaving intertwined vortical structures in the wake.

It could be expected that LEV formation and its spanwise growth might be affected by the presence of the insect body at the centre of rotation. The central body causes the wing root to offset from the axis of rotation by an amount (*b*_0_ = *d*/2), where *d* is the diameter of the body. The body size (both length and diameter) of adult insects vary over a large range. The length, for example, of Hymenoptera, a large order of insects with membrane wings, varies from 0.15 to 60 mm and that of Coleoptera, or beetles, varies from 0.25 to 180 mm [10]. The maximum size of their bodies is limited by their individual tracheal respiration systems [11]. The wings grow mostly after the body is fully grown and the wing size adjusts to the body size [12].

The weights of insects directly depend on their body sizes, requiring different lift forces for flight. A higher lift may be obtained with a larger wing area and a change in wing kinematics. This causes a significant change to the Reynolds number, which affects the near-field and far-field vortex structures, as shown by Liu & Aono [13]. Even the fully grown fruit flies of four different species have different sizes and weights, and fly at four different Reynolds numbers in the range [70 < *Re* < 270] [14], which in terms of our definition of *Re* (equation (2.1)) is approximately [150 < *Re*_*R*_ < 550]. The Reynolds number has been observed to be a significant factor affecting the LEV structure [3,8,9]; this is also confirmed in the present study. However, the focus of the present study is on the change to the LEV structures with body sizes at a chosen Reynolds number. Different insects of similar weights may fly at the same Reynolds number. Their wings could be offset by different amounts depending on their body sizes relative to their wings. The relative change in the Coriolis acceleration can be studied by observing the offsets normalized with the wing spans, instead of studying the overall body sizes.

**Figure 2.**
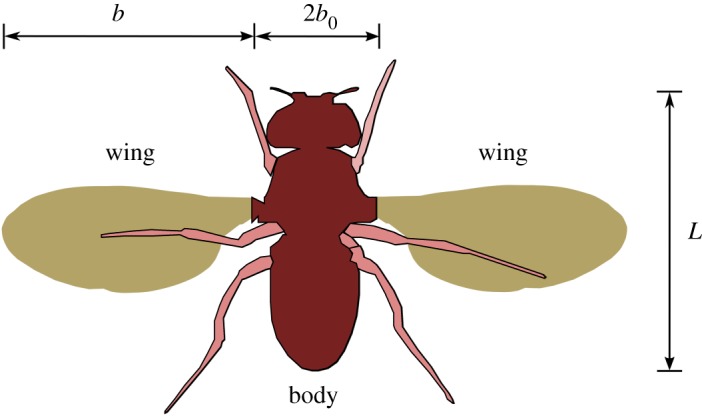
The schematic of a fruit fly (*Drosophila melanogaster*) shows its central body size with respect to the wing size.

The insect body and wing dimensions are shown by a schematic in figure 2. Here, a fruit fly (*Drosophila melanogaster*) wing has been chosen as a representative wing as it has been extensively studied by many researchers both numerically and experimentally [3,13,15–17]. This wing planform is kept constant throughout the experiments to isolate the effects of the wing shape.

The insect dimensions can be normalized using chosen length scales. Ellington [18] has reported such normalized lengths and diameters for a wide range of insects. Thus, the *normalized offset*, which is defined as the ratio of the offset and the wing span (${\hat{b}}_{0}=b_{0}/b$), can be calculated for all these insects, as shown in table 1. For a wide range of insect species, the normalized offset values are found in the range ($0.035<{\hat{b}}_{0}<0.14$). An increase in the offset changes the radius of gyration (*R*_*g*_), resulting in a change in rotational acceleration terms acting across the wing. This may affect the stability of the LEV.

**Table 1.** The data for normalized mean insect body lengths ($\hat{L}$) and mean insect body diameters ($\hat{d}$) are obtained from Ellington [18] and the normalized offsets (${\hat{b}}_{0}$) are calculated using the data.

species	$\hat{L}=\frac{L}{b}$	$\hat{d}=\frac{2b_{0}}{L}$	${\hat{b}}_{0}=\frac{\hat{L}\hat{d}}{2}$
**Coleoptera**			
*Coccinella*	0.73	0.26	0.095
**Diptera**			
*Tipula obsoleta*	0.85	0.11	0.047
*T. paludosa*	1.04	0.10	0.052
*Episyrphus*	1.10	0.16	0.088
*Eristalis*	1.22	0.20	0.122
**Hymenoptera**			
*Apis*	1.62	0.17	0.138
*Psithyrus* and *Bombus*	1.47	0.18	0.132
**Lepidoptera**			
*Emmelina*	0.78	0.12	0.047
*Manduca*	0.81	0.16	0.065
**Neuroptera**			
*Pterocroce*	0.77	0.10	0.039
*Chrysopa*	0.68	0.12	0.041

The effects of the change in the insect body size relative to its wings do not appear to have been studied, although there are some numerical studies showing the effects of the presence of the insect body. Lee *et al.* [19] and Liu *et al.* [20] observed the differences between force production on model wings with and without an insect body, and concluded that the total lift production increased in the presence of the insect body. Wan *et al.* [21] investigated cicada aerodynamics in forward flight and observed that the vortex generated from the cicada thorax enhanced the overall lift. Bomphrey *et al.* [22] performed the smoke flow visualizations and particle image velocimetry (PIV) analysis of the LEVs over the wings of real insects flying in a wind tunnel. They identified different LEV topologies for different insects.

The presence of a central body is unavoidable in experimental models. The central shaft and a connecting rod cause the wing to be offset, resulting in a change in the Rossby number (*Ro* = *R*_*g*_/*c*). Lentink & Dickinson [23] have analysed the Navier–Stokes equation in a non-inertial rotating frame of reference and shown that the Rossby number could influence the centripetal and Coriolis acceleration terms which are responsible for stabilizing the LEVs. Wolfinger & Rockwell [24] have systematically varied the radius of gyration and found that the vortex system degraded rapidly with an increase in the Rossby number, reflecting a change to the relative influence of the rotational acceleration to other force components. Recently, Tudball-Smith *et al.* [25] have observed a variation in the lift coefficients for a rotating wing over a wide range of Rossby numbers (0.7 < *R*_*o*_ < 9). They confirmed that the flow over the wing of a high *R*_*o*_ approached a near-symmetric flow of a translating wing. However, in the experimental studies, the central body size was not changed, whereas the radius of gyration was changed by varying the length of the connecting rod. Moreover, in most of these studies, since the connecting rod was attached to the centre of a rectangular wing's root, the LEV might be unaffected by the secondary flow.

In the aspect ratio studies, for example, Kruyt *et al.* [26] observed an optimum performance for the wing with A∼4, whereas Garmann & Visbal [27], Carr *et al.* [28] and Luo & Sun [29] observed a negligible effect of A on the forces. In these studies, the radius of gyration was also changed with a change in aspect ratio, since the offset and the chord were maintained to be constant. The discrepancies regarding the influence of A were explained later by Lee *et al.* [30] by studying the A–*R*_*o*_ coupling.

Recently, Phillips *et al.* [31] have studied the three-dimensional (3D) LEV structures on the flapping petiolate wings by extending the root of a rectangular wing from its flapping axis. The petiolation has been calculated as the ratio of the wing-offset to the wing-chord (*P* = *b*_0_/*c*). They observed that the LEV's size and strength increased with the petiolation. Interestingly, by identifying the footprints of the LEV near the wing surface, they noted that the LEV was larger at midspan and inboard regions for a longer petiolation. However, the predicted LEV circulatory lift values were shown to increase with petiolation in contradiction to the numerical prediction by Lee *et al.* [30] and Tudball-Smith *et al.* [25] who reported a decrease in the lift with the increasing petiolation. Like the other experimental studies, this study also involved a uniform central body with a changing connecting rod length to extend the offset.

**Figure 3.**
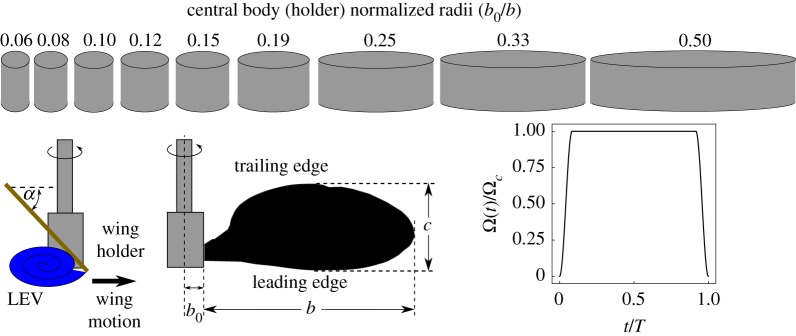
The different holder sizes allow the normalized wing offset to vary in the range ($0.063<{\hat{b}}_{0}<0.5$). The wing was inverted to point the leading-edge downward so as to allow the laser sheet projected from the bottom to illuminate the LEV. The chart on the right shows the angular velocity profile in time. Here, the instantaneous angular velocity *Ω*(*t*) is normalized by the constant angular velocity reached after *t*/*T* = 0.085.

In the present study, the effects of a change in the central body size on the flow structure is systematically studied. Central bodies of different sizes are chosen such that the normalized offset varies in the range ($0.063<{\hat{b}}_{0}<0.5$), which overlaps and extends beyond the offset-ratio range of most insects. The offset values scaled with the wing-chord vary in the range (0.18 < *b*_0_/*c* < 1.45). Figure 3 shows a schematic of different holder sizes with respect to the wing. As indicated, the wing shape is chosen based on a generic fruit fly wing planform, which is kept constant throughout the range of studied offset ratios and Reynolds numbers (600 < *Re*_*R*_ < 3000). Although this range does not include the Reynolds number for *D. melanogaster* owing to the experimental limitations, it includes a larger fruit fly species, *Drosophila mimica*, and we note that different insects such as honeybees and bumblebees have similar wing aspect ratios as that of the chosen wing [14,32,33].

In the present study, the LEV structure for the rotating wing attached to different bodies is obtained by performing scanning PIV experiments, as discussed in §2.3. Unlike insects, the central body in experiments also rotates with the wing. Owing to the limitations of attaching the wing holder to the rotating shaft in experiments, a separate numerical study is conducted with and without the rotation of the wing holder. As discussed in §3.2, this study shows no significant effect on the LEV structure between the two cases.

The LEV is responsible for the low pressure region created on the suction surface of the wing. The pressure distribution on this surface depends on the LEV structure. The split that can occur in the LEV is a prominent feature that can affect the suction. Hence, in the experiments, the difference between the LEV structures for a range of body sizes and Reynolds numbers is observed by comparing the split location of the dual-LEVs, using a similar method to Harbig *et al.* [9]. Even though the LEV can be thought to be influenced by the change in the secondary flow near the wing root and also by the change in the rotational accelerations with the change in offset, at a given Reynolds number, a negligible effect on the LEV-split is observed in the present experiments for the offsets ${\hat{b}}_{0}\leq 0.25$. Interestingly, the vorticity in the secondary vortex weakens with an increase in the offset and eventually, for the larger offsets, the secondary vortex does not split from the primary LEV. Coincidentally, the range of offsets showing a minimal effect on the LEV-split coincides with the range of offsets for most insects (${\hat{b}}_{0}\leq 0.14$).

## 2. Methodology

### 2.1. Experimental facility

The experiments were conducted on a *D. melanogaster* wing planform rotating in a water tank of size 500 × 500 × 500 mm^3^. The wing was cut from a 1 mm thick stainless steel sheet. The wing was held using a central holder attached to the central driving shaft, as shown in figure 3. The mounting was such that the wing root was offset from the central axis by an amount equivalent to the radius of the wing holder (*b*_0_). The wing span (*b* = 120 mm) is measured from the wing root to the wing tip, whereas the chord (*c*) is measured from the leading edge to the trailing edge. The wing aspect ratio is defined as the ratio of the wing span to the mean chord (A = *b*/*c*_*m*_). In the present experiments, A was set equal to 2.91, matching a real fruit fly wing according to Zanker & Götz [16]. The angle made by the wing plane with the horizontal is known as the angle of attack (*α*). In experiments, the leading edge was pointing downwards. This allowed the laser plane to be projected from the bottom of the tank, to be able to illuminate the LEV.

The rotational speed of the wing-shaft was reduced by a belt-and-pulley system by the amount 4.5 : 1. The rotational motion was driven by a brush-less DC motor (model: *EC*-max 30, Maxon Motor) equipped with an encoder (model: ENC24 2RMHF, Maxon Motor) with 5000 counts per turn, and a gear box (model: Koaxdrive KD 32, Maxon Motor) to further reduce the rotational speed by 63 : 1. This reduction also helped in providing sufficient torque to drive the model. The rig was mounted in such a way that the wing shaft was positioned at the centre of the tank.

### 2.2. Wing motion

During the experiments, a simplified motion was prescribed to the wing in order to obtain the LEV structure that is formed during the mid-stroke of a typical flapping cycle of an insect. This required the wing to be held at a constant angle to the horizontal (*α* = 45°) and rotated with a constant angular velocity (*Ω*) without requiring the wing-flip and stroke-reversal. In this study, the wing, with a central body, was rotated through one complete rotation about the central body axis in time *T*. The wing was initially uniformly accelerated over a time of Δ*t* = 0.085*T*. This acceleration period was chosen as the impulsively started wing has been shown to be comparable to the beginning of the downstroke of a flapping cycle [34], with the acceleration period typically ranging between 6 and 10% [3,23]. After this acceleration, the wing reached a constant angular velocity corresponding to the chosen span-based Reynolds number given by

2.1 $$Re_{R}=\frac{U_{g}R}{\nu }.$$

Here, *U*_*g*_ is the velocity at the radius of gyration (*U*_*g*_ = *R*_*g*_*Ω*), *R*_*g*_ is the radius of gyration of the wing, *R* is the total wing span (*R* = *b* + *b*_0_), and *ν* is the kinematic viscosity of water. The wing was decelerated over the last 0.085*T* and stopped. The wing velocity profile chart is shown in figure 3.

The flow field was captured using a scanning PIV technique at the fixed phase angle of *ϕ* = 270°. This phase angle was chosen to allow the flow to reach a near asymptotic (steady) state without running into its own wake. In practice, as demonstrated in §3.1, the flow pattern does not vary significantly between 90° and 315° for moderate Reynolds numbers. The flow in the tank was disturbed by the wing rotation during each experiment. Hence, it was allowed to dissipate the residual vorticity for 10 min before starting the subsequent recording. This was found to be a near-optimal waiting time. A longer time could result in thermal convection driven by a small temperature difference between the fluid and the surroundings.

The effect of the central body size on the flow was investigated by varying the wing holder diameter from the set 2*b*_0_∈ {15, 20, 25, 30, 35, 45, 60, 80, 120} mm, noting again that the holderless wing span was 120 mm. The corresponding radii of gyration were calculated to be *R*_*g*_∈ {75.4, 77.6, 79.9, 82.1, 84.4, 89.0, 96.0, 105.5, 124.6} mm. The LEV structure was investigated over the Reynolds number range 600 < *Re*_*R*_ < 3000. The experiments with the two largest body sizes were conducted in a larger tank of size 1 × 1 × 0.8 m^3^ with the body located at the centre of the tank. Note that even for the smaller tank, the cross-sectional blockage ratio of the wing is only approximately 2%.

### 2.3. The scanning particle image velocimetry system

The LEV was observed to have a 3D structure with vorticity aligned predominantly in the spanwise direction. Hence, the structure of the LEV could be characterized by obtaining the spanwise vorticity field in cross-sectional planes at multiple spanwise locations of the wing. This would require the laser apparatus, or at least the laser sheet, to be shifted to different locations for every recording. Considering an idle time of 10 min between two recordings, obtaining PIV images at different spanwise locations with standard PIV could be a time-consuming process.

Researchers in the past have developed scanning PIV systems for fast recording of multiple planes within a volume. For instance, Brücker [35], David *et al.* [36], and Lawson & Dawson [37] used an oscillating mirror, whereas Green *et al.* [38] and Albagnac *et al.* [39] used a mirror mounted on a high-speed linear traverse to scan the volume. David *et al.* [36] performed scanning PIV measurements for an aerofoil of a finite aspect-ratio flapping in two dimensions (linear translation and pitch).

**Figure 4.**
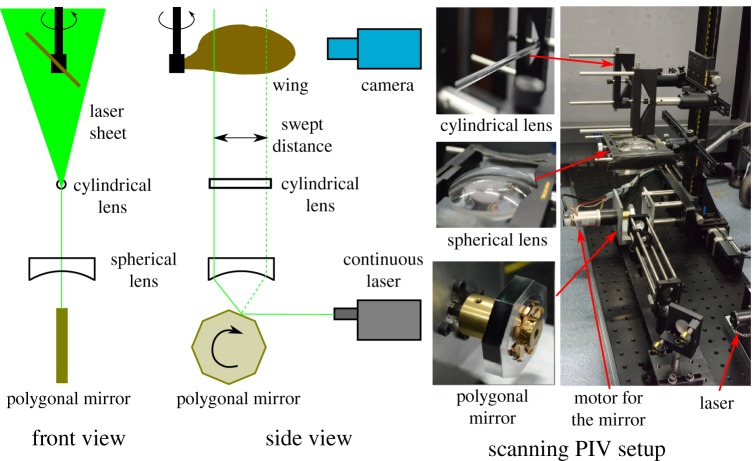
Schematic of the scanning PIV system is shown in the front and side views. As the polygonal mirror rotates, the laser sheet scans the distance in a spanwise direction. The pictures on the right show the scanning PIV setup and its components.

In the present work, a scanning PIV system was developed with a rotating mirror. It allowed multiple images to be taken at high speed during a rotation of the wing. It also provided a scanning resolution of about Δ(*r*/*b*) = 0.025 in the spanwise shift, where *r* is the distance along the span measured from the wing-root. A schematic of this scanning PIV setup is shown in figure 4. A laser beam from a continuous laser (model: MLL-N-532nm-5W, CNI) is reflected by a polygonal (octagonal) mirror. The reflected beam passes through a plano-concave spherical lens. The spherical lens is aligned such that the beam is refracted only in the vertical direction. This is followed by a cylindrical lens that forms a laser sheet. The laser sheet illuminates the PIV particles (model: S-HGS-10) around the wing in the fluid.

The rotation of the polygonal mirror was achieved using a Maxon *EC*-max 30 servo motor and a Maxpos 50/5 position controller. As the polygonal mirror rotated, the laser sheet shifted its position along the wing span. PIV images were obtained at different spanwise positions using a PCO Dimax S4 camera sampling at a rate of 1000 frames per second (FPS). An exposure of 0.75 ms was set so as to limit the thickness of the shifting laser within 3 mm. With this exposure, the particle blur was less than one pixel even for the wing with a large offset (${\hat{b}}_{0}=0.5$) rotating at a high Reynolds number (*Re*_*R*_ = 3000).

For a single set of PIV measurements, the polygonal mirror was rotated through 720°. The mirror had eight reflecting faces. Each mirror face could reflect the beam for 45° rotation of the polygon. The camera was triggered only for a 15° rotation range of each face when the laser plane fell in the volume of interest. During the remaining 30°, the laser plane was shifted out of the volume of interest. Overall, 16 such scanning sets were obtained during the measurements, with each set containing 26 images. Only the central four scanning sets were processed to obtain three sets of PIV image pairs used to reconstruct the velocity field. Scanning PIV was performed in the range 600 < *Re*_*R*_ < 1500. During the recording time of the central four sets, in the worst case (*Re*_*R*_ = 1500), the wing rotated through 269.45°–270.55°. The LEV structure remained unchanged for a wide range of phase angles, as described in the results. For higher Reynolds numbers (*Re*_*R*_ > 1500), the fixed-plane PIV was performed.

**Figure 5.**
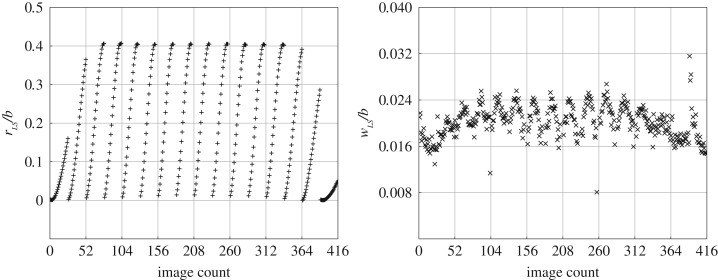
The scanning motion of the laser plane is tracked from the side view. The position of the laser plane, normalized by the wing span (*r*_*LS*_/*b*), is tracked in the successive images of a scan. The system is capable of scanning *r*_*LS*_/*b* = 0.4 across the span consistently, except for the first and last three cycles, where the rotating mirror is accelerating or decelerating. The normalized laser sheet width (*w*_*LS*_/*b*) is consistently between 0.016 and 0.025.

In order to calibrate the laser-plane position with respect to the mirror rotation, images were obtained from the side view. Figure 5 shows the results of the laser-plane position and the corresponding laser-sheet width varying across the imaging sequence. Both the quantities are normalized by the wing span to compute the normalized position (*r*_*LS*_/*b*) and normalized width (*w*_*LS*_/*b*) of the laser sheet. From the figure, it can be seen that the laser sheet is able to consistently scan through a distance of *r*_*LS*_/*b* = 0.4 except for the first and last three scans. This is because when the mirror accelerates or decelerates, the distance travelled by the laser sheet in the chosen amount of time is less than that at the constant rotation speed programmed. The laser sheet width observed is in the range 0.16 ≤ *w*_*LS*_/*b* ≤ 0.25 (i.e. approximately between 2 and 3 mm) for most images. The laser plane position in a particular scan can be identified within an accuracy of 1 mm by the index of the image in the scan.

For PIV measurements, the camera was focused at the centre of the scanned distance. The aperture opening was set to *f*/8 to provide a greater depth-of-field. Images of the same index from two consecutive scans were paired. Cross correlation was performed between the pairs using in-house codes originally developed by Fouras *et al.* [40]. An interrogation window of 32 pixels × 32 pixels with 75% overlap yielded a 198 × 249 array of velocity vectors. The number of bad vectors in the cross correlation was more for the out-of-focus images. The PIV images sampled in the middle portion of the scan, equivalent to a normalized distance Δ(*r*_*LS*_/*b*) = 0.25, resulted in a total number of bad vectors below 5%.

To reconstruct the two-component velocity field over the entire wing span, the optical components were shifted to different positions along the span. The camera focus was readjusted and the scanning PIV measurements were repeated at the new positions. This was achieved by mounting the optical components on a motorized linear traverse (model: Zaber T-LSR450) controlled using Zaber Console software.

**Figure 6.**
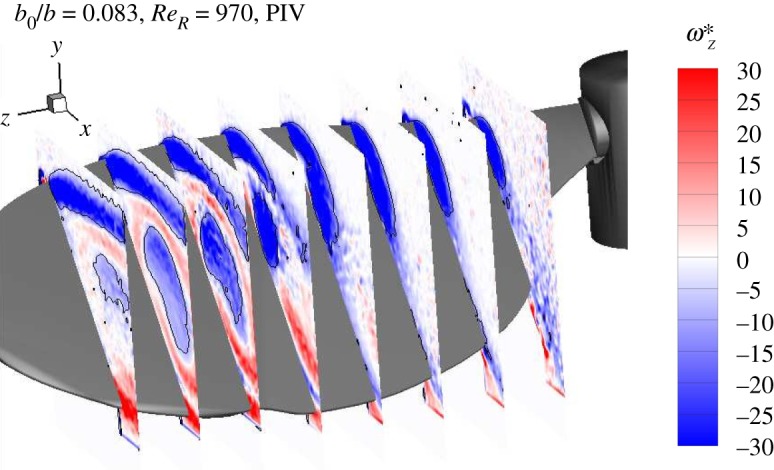
Vorticity plots at different spanwise locations, obtained from the scanning PIV are plotted. The normalized vorticity range is *ω*^⋆^_*z*_ = *ω*_*z*_*R*/*U*_*g*_∈[ − 30, 30]. The black lines represent the vortices identified by the *Q* criterion.

At a fixed position of the traverse, scanning PIV measurements were undertaken to produce three full independent datasets. For each set, three central consecutive scanning cycles were chosen from 16 cycles, as discussed earlier. Thus, nine sets of PIV images for each spanwise position were obtained. The two-dimensional planar velocity fields were computed separately for each set by cross-correlation. Finally, the flow quantities were averaged to give a mean flow field within each spanwise plane for each scanned volume. Figure 6 shows the evolution of typical LEVs through averaged vorticity contours obtained from the scans, where the vortex structures are identified by the *Q*-criterion [41] ignoring the unmeasured through-plane velocity component. The LEV-evolution and its reconstruction from the isosurface of constant *Q*-criterion can be found in the electronic supplementary material (https://figshare.com/s/f54a4c4a32eae9bf6ebf). It can be seen that the flow separates at the leading edge, forming the characteristic LEV. The LEV grows in size with increasing *r*/*b*. The wing, camera and the motor for the scanning laser-sheet system were controlled by a real-time control system described in the following section.

### 2.4. Real-time control system

A real-time control system with high temporal accuracy was required to accurately control and trigger such a complicated experimental rig consisting of a high-speed camera for PIV imaging and motors for driving the wing model and the rotating mirror. This was achieved by developing a system based on EtherCAT (Ethernet for Control Automation Technology). In this system, the motor controllers (model: Maxpos 50/5, Maxon Motor) and the digital I/O modules for the triggers were connected to an EtherCAT coupler (model: EK1100, Beckhoff). The coupler was connected via an Ethernet cable to a high-performance workstation computer that was equipped with the Beckhoff TwinCat3.1 software providing a real-time accuracy of 0.125 ms. Customized graphical user interface programs were developed to control the real-time tasks.

### 2.5. Computational setup

The effect of the holder with and without the rotation was investigated numerically. The wing and holder geometries, modelled numerically, were same as in the experiments. The flow over the rotating wing was modelled by combining the Navier–Stokes equations in a non-inertial reference frame and the continuity equation:

2.2 $$\frac{\delta \rho u_{\mathrm{abs}}}{\delta t}+\nabla \cdot (\rho uu_{\mathrm{abs}})=-\nabla p+\nabla \cdot \tau -2\rho \Omega \times u-\rho \Omega \times (\Omega \times r)$$

and

2.3 ∇⋅u=0,

where *ρ* is the fluid density, ***u*** and ***u***_*abs*_ are the velocity vectors in rotating and absolute frames, *p* is the pressure, ***τ*** is the stress tensor, ***Ω*** is the rotational velocity and ***r*** is the location vector. The commercial finite-volume-based code ANSYS CFX was employed to solve the equations using direct numerical simulations. The wing span was chosen as 2.47 mm and a holder was scaled according to the wing span to match the offset ratio (*b*_0_/*b* = 0.25) as in the experiments. The details of the computational domain and the mesh validation can be found in [9]. The non-rotating holder was also modelled by specifying the fluid velocity at the wall in the rotating frame.

The wing was kept at a constant angle, *α* = 45°. The motion profile of the wing was maintained to be the same as the experiments, with a steady acceleration over a period (0.085*T*) followed by a constant rotational velocity. This was simulated until the wing completed 270° of rotation. The lift and drag coefficients were calculated as *C*_*L*_ = 2*L*/*ρU*^2^_*g*_*bc*_*m*_ and *C*_*D*_ = 2*D*/*ρU*^2^_*g*_*bc*_*m*_, respectively.

## 3. Results

### 3.1. Effect of phase angle

Initially, the LEV formation and its overall variation with the phase angle of wing rotation (*ϕ*) were investigated experimentally. The wing, with an offset ratio ${\hat{b}}_{0}=0.08$, was rotated with a constant angular velocity corresponding to a Reynolds number of *Re*_*R*_ = 900, and PIV images were recorded at different phases in steps of 45°.

**Figure 7.**
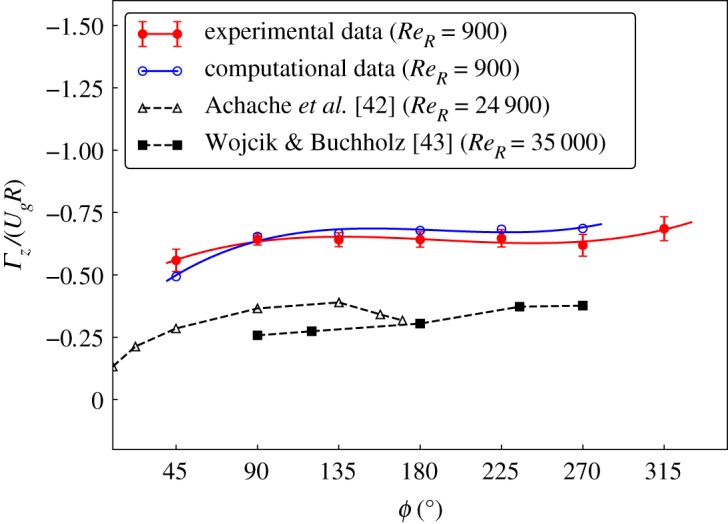
As the wing rotates, the LEV is found to grow in size in the initial phases and then remains stable. The normalized circulation (*Γ*_*z*_/*U*_*g*_*R*) increases initially until *ϕ* = 90° and remains unchanged until *ϕ* = 270°. The development of circulation is compared to the numerical predictions with a similar geometry. The results are also compared to the results at high *Re*_*R*_ reported by Achache *et al.* [42] for a hummingbird wing (A = 3.35) rotating with *α* = 30° and those by Wojcik & Buchholz [43] for a rectangular wing (A = 4) with *α* = 35°, both at *z*/*R* = 0.5.

Figure 7 shows the evolution of the LEV as the wing is rotated. The prominent vortical structure identified as the LEV is visualized through the spanwise vorticity field. The LEV is initially compact at *ϕ* = 45° and then increases in size. Beyond *ϕ* = 135°, it maintains a constant size and strength over most of the rotation cycle. However, clearly the LEV size changes towards the end of the rotation period, possibly owing to interference with residual vorticity generated at the start of the rotation. The generally accepted reason behind the stable size of the LEV, despite it being continuously fed circulation from the separating shear layer, is the stable rotational acceleration, as described by Lentink & Dickinson [23].

The stability of the LEV can be shown quantitatively by calculating the circulation in the area around the LEV and examining how it changes over the rotation. The circulation of the LEV is calculated using the method described in §3.3. The development of the circulation with the phase angle (*ϕ*) can be seen in figure 7. The initial increase in the normalized circulation (*Γ*_*z*_/*U*_*g*_*R*) observed during the acceleration (*ϕ* < 90°) has also been reported by Achache *et al.* [42] and Elimelech *et al.* [44]. The circulation remains unchanged during the rotation phase 90° < *ϕ* < 270°. This observation is consistent with the stable lift observed in the same range in experiments by Birch *et al.* [3] (for *α* = 40°) and numerical simulations by Harbig *et al.* [9] (for *α* = 45°). The normalized circulation values predicted numerically by simulating the same geometry are observed to be close to those observed in experiments, with a small variation (<10%). A comparison with the circulation obtained by Achache *et al.* [42] and Wojcik & Buchholz [43] also showed a relatively stable circulation post *ϕ* = 90°. The values from both the studies have been scaled as per the present method of normalizing the circulation. It should be noted that the overall lower values in both the cases, compared to the present results, may be because of their higher wing aspect ratios, owing to the fact that the circulation reduces significantly with an increase in A [9]. The lower values of *α* and differences in the wing shapes in both compared to the present case could be the additional factors that may have caused this reduction in circulation. The drop in the normalized circulation post *ϕ* = 135° observed by Achache *et al.* is owing to the deceleration of the wing.

### 3.2. Effect of holder rotation

**Figure 8.**
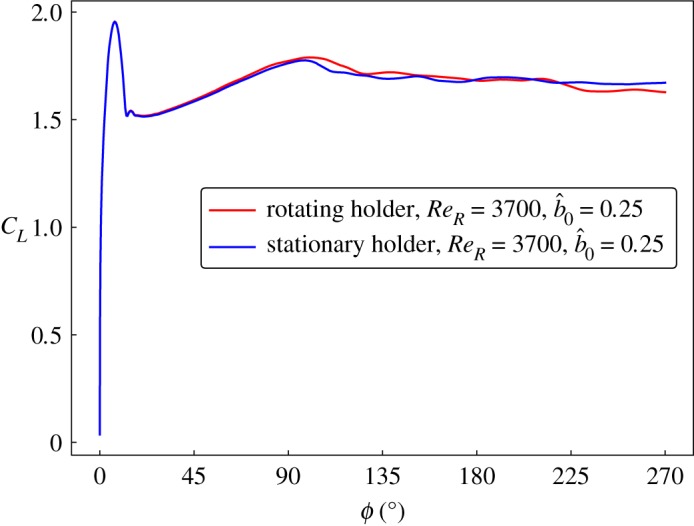
The lift coefficients on the wing for a large holder (*b*_0_/*b* = 0.25) with and without its rotation remain mostly unchanged even at a large Reynolds number (*Re*_*R*_ = 3700).

Since it is difficult to maintain a stationary central body in experiments, the effect of its rotation was studied by simulating the wing rotation numerically with and without the motion of the holder. The two conditions were compared by observing the lift coefficients on the wing. A large holder (*b*_0_/*b* = 0.25) and a large Reynolds number (*Re*_*R*_ = 2910) were selected to accentuate possible differences. As can be seen in figure 8, there is a negligible difference in the lift coefficients in the two cases with the rotation of the wing. Thus, the experiments with a rotating holder can be assumed to have the same effects as that of a stationary central body.

### 3.3. Determining the leading-edge vortex characteristics

**Figure 9.**
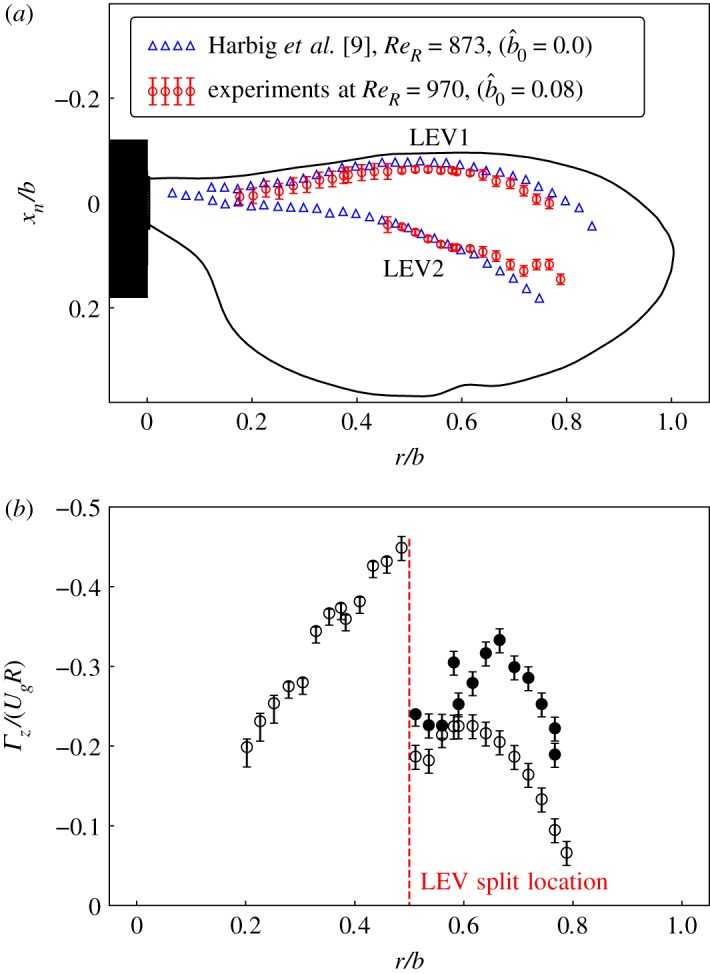
In (*a*), the LEV centres identified using the local peak of *γ*_1_ from PIV images are plotted along with those reported by Harbig *et al.* [9]. The LEV split was identified by the sudden drop in the sectional circulation around the LEV region (|*γ*_2_| > 2/*π*) as seen in (*b*). The open symbols represent the LEV close to the leading edge, whereas the filled symbols represent the secondary LEV split from it.

The datasets obtained from the scanning PIV at different spanwise planes, for *ϕ* = 270°, were stitched together to build a 3D view of the LEV. The LEV centre was identified by the local maximum of the scalar *γ*_1_ using the criterion of Graftieaux *et al.* [45]. Here, *γ*_1_ was calculated from the PIV data at each spanwise interval as:

3.1 $$\gamma_{1}=\frac{1}{N}\sum_{S}\frac{(PM\wedge U_{M})\cdot z}{||PM||\cdot ||U_{M}||},$$

where M is any point in an area *S* around point P, *z* is the unit normal vector, *U*_*M*_ is the velocity vector at M, and *N* is the number of points M inside *S*. *γ*_1_ is equivalent to the ensemble average of the term sin(*θ*_*m*_), where *θ*_*m*_ represents the angle between the radius vector *PM* and the velocity vector *U*_*M*_. The locations of the vortex centres identified on different spanwise planes are plotted in figure 9*a*. In this figure, two different LEV centres can be seen. LEV1 remains close to the leading edge while LEV2 moves inward. The LEV centres identified from PIV images show a good match with the numerical predictions of Harbig *et al.* [9].

An examination of the vorticity contours in figure 6 shows that as the distance between the leading edge and LEV2 increases and LEV2 grows in size, there exists a region of positive vorticity beneath LEV2, kinematically generated by the induced velocity gradient at the surface to satisfy the no-slip boundary condition. With an increase in *r*/*b* and an increase in LEV2 circulation, this region of positive boundary layer vorticity grows in size, diffuses away from the boundary, and is advected clockwise around LEV2. As this secondary vorticity moves towards the separating shear layer it will weaken and effectively sever it, stopping vorticity being fed into LEV2 and allowing LEV1 to form a separate structure. Lu *et al.* [8] provide further discussion of this splitting process.

The location of the split can be quantified by computing the circulation of the LEVs. The circulation of the LEV structure was calculated using the field *γ*_2_ of the vortex core identification algorithm of Graftieaux *et al.* [45]:

3.2 $$\gamma_{2}=\frac{1}{N}\sum_{S}\frac{[PM\wedge (U_{M}-U_{P})]\cdot z}{||PM||\cdot ||U_{M}-U_{P}||}.$$

The region with |*γ*_2_| > 2/*π* represents the flow locally dominated by rotation. The circulation of the LEV was calculated by integrating the vorticity inside this identified region. The normalized circulation (*Γ*_*z*_/*U*_*g*_*R*) was observed to increase as we move away from the wing root. At a certain spanwise location, the circulation drops and shows two values corresponding to the dual-LEVs. This location is identified as the LEV-split location, as can be seen in figure 9*b*.

### 3.4. Effect of *Re* and presence of a central body

**Figure 10.**
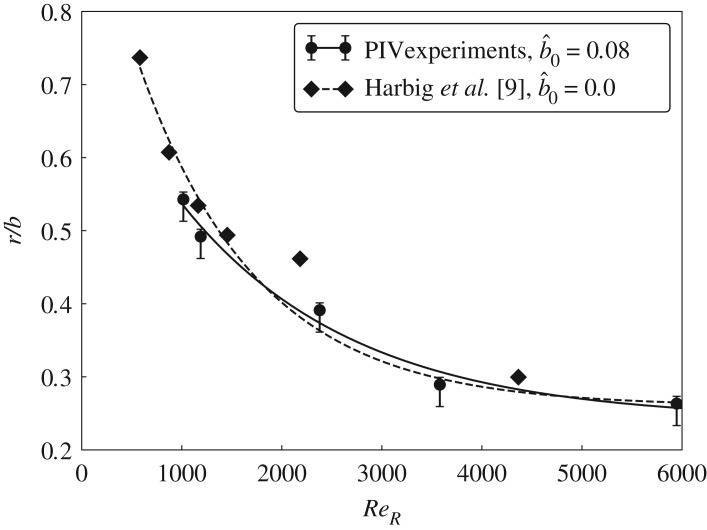
The normalized spanwise location (*r*/*b*) for the LEV split is shown as a function of *Re*_*R*_. The experimental data are for the wing with a central body and the computational data by Harbig *et al.* [9] are for the wing without a central body and no offset.

An overall increase in the body size of an insect causes an increase in its mass and the Reynolds number. The effect of *Re* on the LEV structures was studied by tracking the change in the LEV-split location. Lu *et al.* [8] have reported that the dual-LEVs could be observed only for the chordwise Reynolds numbers *Re* > 640. However, we observed the dual-LEVs even at *Re*_*R*_ = 920 which corresponds to the chordwise Reynolds number *Re* = 290. Even though the existence of dual-LEVs was reported by Lu *et al.* [8] and Carr *et al.* [28], the split location was found to be tracked with *Re* only by Harbig *et al.* [9]. Hence, to provide a level of validation, the experimental results are compared directly with the numerical predictions of Harbig *et al.* [9]. The wing geometry and kinematics are identical for this comparison. The only difference is that the numerical study does not model the central holder that is present in the experiments. The Reynolds number range investigated was 920 < *Re*_*R*_ < 8750. The split location normalized by the wing span (i.e. *r*/*b*), is plotted as a function of *Re*_*R*_ in figure 10.

The present results and those of Harbig *et al.* [9] show the same trend, with the split location shifting radially inwards as the Reynolds number increases. It should be noted that in the original analysis to determine the variation of the split location with Reynolds number, Harbig and co-workers used the standard Q-criterion including the spanwise velocity component. That leads to slightly increased outward split positions, and thus their data were reanalysed here to use the same criterion as in these experiments, i.e. still using the Q-criterion but neglecting terms involving the spanwise velocity component that was not measured in the experiments. Clearly, figure 10 shows that the experimental and computational split locations match to within a few per cent over the Reynolds number range considered, despite the presence of the 10 mm radius holder, although noting that it equates to only ∼8% of the wing span.

There may be several reasons behind the inward shift of the LEV split location with an increase in *Re*_*R*_. First, at a high *Re*_*R*_, the reduced viscous effects cause a reduction in the diffusion of the vorticity of LEV2, therefore confining it to a smaller volume and making it more concentrated. This increased vorticity of the LEV in proximity to the wall would induce stronger secondary positive vorticity at the surface beneath LEV2. This vorticity is then advected more strongly around the LEV at higher *Re*_*R*_, because the velocity closer to the surface will be higher, to interact with the leading edge separating shear layer, as discussed previously. This process may cause the LEV structure to separate and split at a more inward location than that at a lower *Re*_*R*_. Second, the increased turbulence at even higher *Re*_*R*_ may make the LEV structure more unstable and prone to the split at a more inboard location. Therefore, the movement of the LEV split towards the wing root at a higher *Re*_*R*_ may be owing to a combination of factors.

However, in general, the presence of the central wing holder causes the wing root to be offset from the axis of rotation, thus affecting the Rossby number, and it also generates secondary flow at the root that may disturb the formation of the LEV. To assess these effects for considerably larger holder sizes, the effect of the central body size on the LEV structure is further investigated in the following section.

### 3.5. Effect of offset ratio

The central holder diameter was varied between 15 and 120 mm maintaining the wing geometry. Hence, the wing root was offset from the axis of rotation, such that the offset ratio varied in the range ($0.063<{\hat{b}}_{0}<0.5$). As there is an eight times change in the offset ratio, it was expected to see a change in the LEV structure. Hence, the flow structure was obtained for different offset ratios, for the wing rotated at different Reynolds numbers (600 < *Re*_*R*_ < 3000). First, a comparison of the LEV structures at a chosen Reynolds number was made for different offsets. The 3D LEV structures were visualized by observing the isosurfaces of the normalized *γ*_2_ = 2/*π* using the data obtained from the scanning PIV.

**Figure 11.**
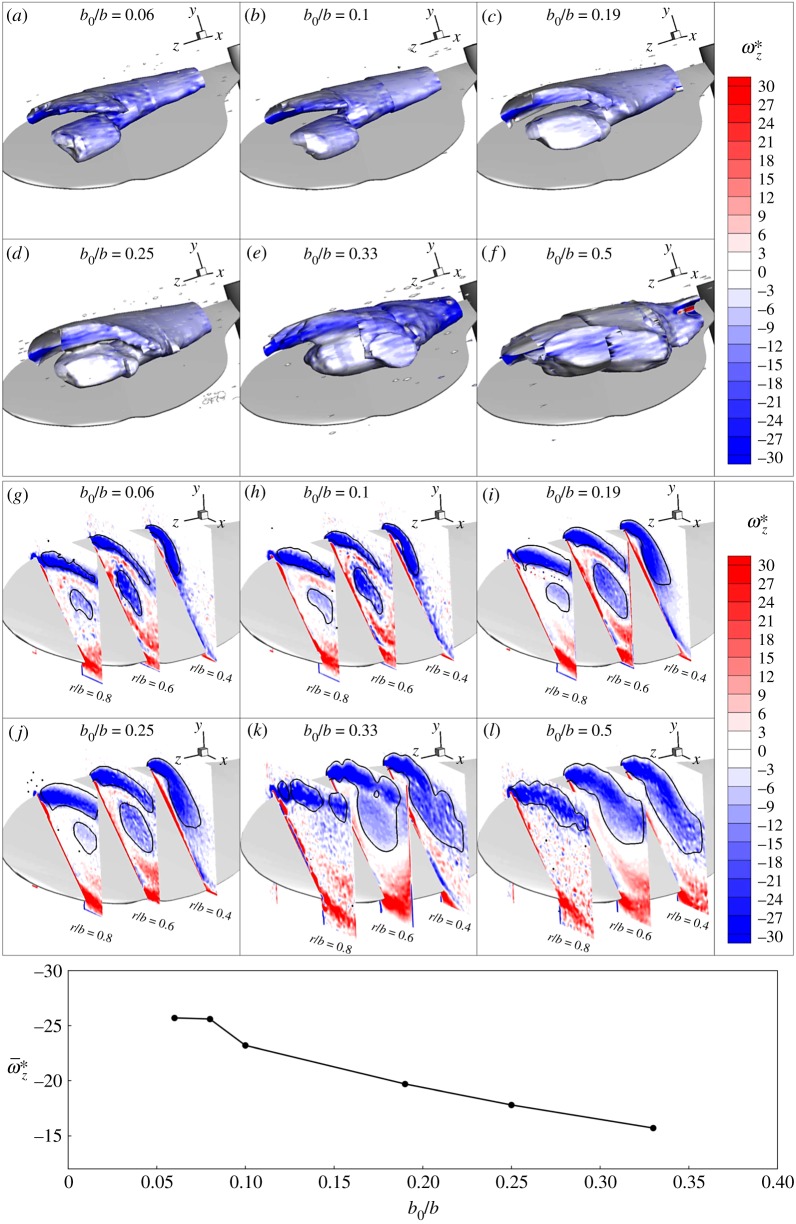
For the wing rotating at *Re*_*R*_ = 1000, the LEV structures for different offsets are obtained from the isosurfaces of *γ*_2_ = 2/*π* from the PIV data at various spanwise locations, shown in subfigures (*a*)–(*f* ). The isosurfaces are coloured with normalized spanwise vorticity. Subfigures (*g*)–(*l*) show the normalized vorticity contours at three spanwise locations (*r*/*b* = 0.4, 0.6 and 0.8) for different offsets. The figure at the bottom shows the mean vorticity inside the secondary LEV at *r*/*b* = 0.6 as a function of the normalized offset.

Figure 11*a*–*f* show a comparison of the LEV structures for the wing rotating at *Re*_*R*_ = 1000 with different holders. A large secondary LEV was observed to split from the primary LEV. However, the difference between the LEV structures did not seem significant for offsets ${\hat{b}}_{0}\leq 0.25$. In figure 11*g*–*l*, the normalized vorticity contours are plotted on different spanwise planes, which show that the LEV split occurred at a similar location for ${\hat{b}}_{0}\leq 0.25$. By comparing the vorticity contours on the spanwise planes at *r*/*b* = 0.6 for different offsets, it can be observed that the secondary vortex after the split has a lower strength with the increasing offset. This reduction in the strength is evident from the reduced mean vorticity inside the secondary LEV with an increase in the offset, as shown in figure 11. Tudball-Smith *et al.* [25] have also observed the reduction in the LEV strength with an increase in Rossby number.

It can be seen in figure 11*g*–*l* that, for lower *b*_0_/*b* values, the regions near the LEV centres LEV1 and LEV2 (as shown in figure 9) contain relatively larger magnitudes of the negative spanwise vorticity. As described earlier, a small region of positive vorticity induced by the LEV is observed to grow in size with an increase in *r*/*b*. At a certain location along the span, this vorticity gets entrained in the LEV structure, forming the dual-LEVs. With an increase in the wing-root offset, the spanwise flow and effects of the Coriolis force are reduced, in line with the reduced streamwise velocity difference between the wing root and tip. The Coriolis force is important for maintaining a compact LEV2 close to the wing surface, as shown by Jardin & David [46]. This change to the LEV structure with offset upstream of the split position is clearly seen in figure 11. This leads to a reduction in the boundary-layer secondary vorticity generated at the surface beneath the LEV structure, and hence a reduced tendency for the LEV structure to split. For very large offsets (such as ${\hat{b}}_{0}=0.33$ and 0.5), this region of positive vorticity is so weak that LEV2 remains attached to the primary shear layer and hence, no clear split is observed. The LEV split location was tracked for ${\hat{b}}_{0}\leq 0.25$ to represent the quantitative comparison of the flow characteristics.

**Figure 12.**
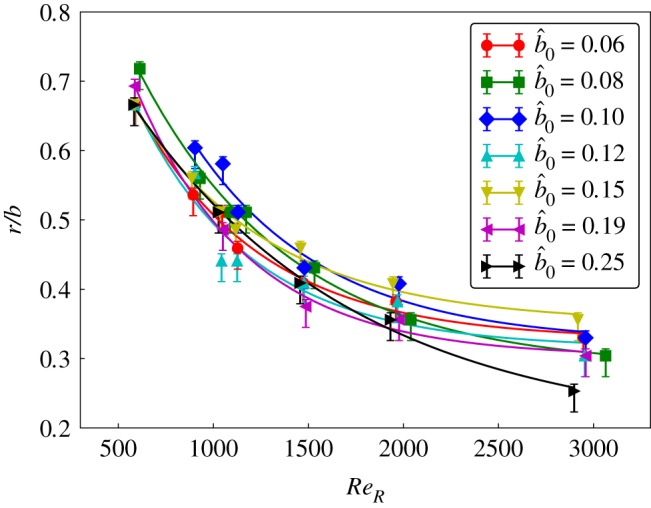
Although the LEV split position exponentially shifts towards the wing root with *Re*_*R*_, it remains in a narrow region for any given *Re*_*R*_, for the offset ratios ${\hat{b}}_{0}\leq 0.25$. The smooth lines represent the exponential fits to the data. The LEV split could not be identified for the offset ratios 0.33 and 0.5.

Figure 12 shows the variation of the LEV split location with Reynolds number for different offsets. It can be seen that regardless of the offset ratio, the split locations are close to each other, which only depends on the Reynolds number. The difference in the split locations at a given Reynolds number is within the uncertainty of the experiments. Since the split position is an important feature affecting the LEV structure, the above results show that, for the range of offset ratios ${\hat{b}}_{0}\leq 0.25$, the central body size has a minimal effect on the LEV structure. In addition, it appears that the change to the spanwise flow onto the wing root induced by different holder sizes does not strongly affect the LEV split. However, it may have affected the strength of the secondary vortex in terms of the spanwise vorticity. The strength of the secondary vortex for the offsets ${\hat{b}}_{0}=0.33$ and 0.5 is so low that it does not separate from the primary shear layer.

## 4. Conclusion

In this study, the effect of the Reynolds number and the central body size on the spanwise position where the LEV splits into dual-LEVs, which is used as a proxy for overall LEV development, is studied experimentally for the flow over a rotating fruit fly wing model. The range of offset ratios investigated includes the offset ratios for most insects.

The structure of the LEVs was obtained using a scanning PIV technique. The central body's rotational motion as compared to the stationary body of insects was found to have a negligible effect. Thus, the scanning PIV measurements were conducted with the wing and the central body rotated at span-based Reynolds numbers between 600 and 1500. The fixed-plane PIV measurements were conducted for 2000 < *Re*_*R*_ < 10000. The dual-LEVs were observed to split at radially inward locations with an increase in the Reynolds number. The comparison of the split locations with those obtained from numerical simulations of an identical wing by Harbig *et al.* [9] showed good agreement for a small central body size. Further experiments using a wide range of body sizes causing the offset ratios to be $0.06\leq {\hat{b}}_{0}\leq 0.25$ showed a negligible effect on the LEV split to within experimental uncertainty bounds. However, the larger offset ratios resulted in a significantly different LEV structure with no clear identifiable split. It can be speculated from the reduced vorticity in the secondary vortex that it may have an influence on the lift and the drag forces on the wing with large offset ratios. This will be investigated in a future study.

It is interesting to note that for most insects, the body sizes create offsets in the range ${\hat{b}}_{0}\leq 0.14$. Thus, despite the influence of a central body on the fluid feeding into the LEV and the relative influence of the rotational acceleration to other acceleration components, over this range of body sizes, the change in Reynolds number has a significant influence. However, the wing offset owing to the change in the central body diameter was found to have a minimal effect in the normal insect range of offset ratios.

## Supplementary Material

LEV reconstruction from scanning PIV data

## Data accessibility

The datasets supporting this article have been uploaded to https://figshare.com/s/8fbb1a2280f172da1664.
